# Combining radiation with autophagy inhibition enhances suppression of tumor growth and angiogenesis in esophageal cancer

**DOI:** 10.3892/mmr.2015.3623

**Published:** 2015-04-16

**Authors:** YONGSHUN CHEN, XIAOHONG LI, LEIMING GUO, XIAOYUAN WU, CHUNYU HE, SONG ZHANG, YANJING XIAO, YUANYUAN YANG, DAXUAN HAO

**Affiliations:** 1 Departments of Radiation Oncology, Zhengzhou University Affiliated Cancer Hospital, Henan Cancer Hospital, Zhengzhou, Henan 450008, P.R. China; 2 Departments of Pathology, Zhengzhou University Affiliated Cancer Hospital, Henan Cancer Hospital, Zhengzhou, Henan 450008, P.R. China; 3 Department of Radiation Oncology, Zhengzhou People’s Hospital, Zhengzhou, Henan 450053, P.R. China; 4 Department of Pathology, Zhengzhou University Affiliated Zhengzhou Central Hospital, Zhengzhou, Henan 450003, P.R. China

**Keywords:** autophagy, apoptosis, radiosensitivity, esophageal cancer, squamous cell carcinoma to enhance the radiosensitivity of esophageal squamous cell carcinoma

## Abstract

Radiotherapy is an effective treatment for esophageal cancer; however, tumor resistance to radiation remains a major biological problem. The present study aimed to investigate whether inhibition of autophagy may decrease overall tumor resistance to radiation. The effects of the autophagy inhibitor 3-methyladenine (3-MA) on radiosensitivity were tested in the EC9706 human esophageal squamous cell carcinoma cell line by colony formation assay. Furthermore, the synergistic cytotoxic effects of 3-MA and radiation were assessed in a tumor xenograft model in nude mice. Mechanistic studies were performed using flow cytometry, immunohistochemistry and western blot analysis. The results of the present study demonstrated that radiation induced an accumulation of autophagosomes and 3-MA effectively inhibited radiation-induced autophagy. Inhibition of autophagy was shown to significantly increase the radiosensitivity of the tumors *in vitro* and *in vivo*. The enhancement ratio of sensitization in EC9706 cells was 1.76 when the cells were treated with 10 mM 3-MA, alongside ionizing radiation. In addition, autophagy inhibition increased apoptosis and reduced tumor cell proliferation. The combination of radiation and autophagy inhibition resulted in a significant reduction in tumor volume and vasculature in the murine model. The present study demonstrated *in vitro* and *in vivo* that radiation-induced autophagy has a protective effect against cell death, and inhibition of autophagy is able to enhance the radiosensitivity of esophageal squamous cell carcinoma.

## Introduction

Esophageal cancer has garnered attention in the past three decades, due to its increasing prevalence and poor prognosis (1). Radiotherapy is an effective treatment option in curing or controlling esophageal cancer. Ionizing radiation kills tumor cells by inducing various types of DNA damage, including double-strand and single-strand breaks, base damage and DNA-DNA or DNA-protein cross-linking (2). However, normal tissue dose constraints and tumor radioresistance are considered major obstacles to the success of radiotherapy. Previous attempts have been made to reduce resistance to radiotherapy and enhance its therapeutic effectiveness. It has previously been shown that local tumor control may be improved when radiation therapy is combined with chemotherapy or thermotherapy (3,4); however, the effect of current therapies in improving the survival of patients with esophageal cancer remains unsatisfactory (5).

Autophagy is an evolutionarily conserved catabolic process, which targets cellular organelles and cytoplasmic constituents to lysosomes for degradation. Numerous studies have indicated the importance of autophagy in the pathogenesis, development and treatment of cancer (6–8). However, the role of autophagy in cancer remains controversial (9,10). A basal level of constitutive autophagy maintains homeostasis and cellular health, through the removal of excess or damaged intracellular components and microbial invaders, thus suppressing cancer initiation and progression (11). However, once cells have turned cancerous, autophagy may aid cancer cell survival through degradation and recycling of unnecessary, injured or aged proteins and organelles of normal cells (12). Therefore, appropriate modification of autophagy, such as inhibition of cytoprotective autophagy, may be an appropriate therapeutic strategy for the treatment of established cancers. The association between autophagy and angiogenesis is complex, and there are various conflicting reports regarding the role of autophagy in the process of angiogenesis. Previous studies have demonstrated that autophagy inhibits angiogenesis (13,14), whereas other studies have suggested that autophagy promotes cancer, and inhibition of autophagy prevents angiogenesis (15,16). However, whether autophagy affects angiogenesis in esophageal cancer remains poorly understood.

A preliminary study demonstrated that inhibition of autophagy enhanced the cytotoxicity of radiotherapy in the TE-1 esophageal cancer cell line (17). The present study further validated these previous observations and also aimed to investigate the correlation between autophagy and tumor angiogenesis using *in vitro* and *in vivo* assays. Mechanistic studies were performed using flow cytometry, immunohistochemistry and western blot analysis, and a xenograft model of esophageal cells was treated with radiation and an autophagy inhibitor followed by histological and western blot analysis. The present study provided proof that the inhibition of autophagy may improve the outcomes of radiation therapy of human esophageal squamous cell carcinoma.

## Materials and methods

### Cell culture

The EC9706 human esophageal squamous cell carcinoma cell line was obtained from the Type Culture Collection of the Chinese Academy of Sciences (Beijing, China). The cells were cultured in RPMI-1640 medium supplemented with 10% fetal bovine serum, 2 mM glutamine, 100 units/ml penicillin and 100 *µ*g streptomycin/ml (all purchased from Sigma-Aldrich, St. Louis, MO, USA). The cells were incubated at 37°C in a humidified atmosphere containing 95% air and 5% CO_2_, and were sub-cultured every three days.

### Reagents and antibodies

Autophagy inhibitor 3-methyladenine (3-MA) was obtained from Sigma-Aldrich. Anti-microtubule-associated protein light chain 3 (LC3; cat. no. sc-16755), anti-beclin-1 cat. no. sc-48381), anti-vascular endothelial growth factor (VEGF; cat. no. sc-1836), anti-cleaved caspase-3 (cat. no. sc-22171-R), anti-cleaved caspase-9 (cat. no. sc-56073), anti-cleaved poly(ADP ribose) polymerase (PARP; cat. no. sc-23461-R), anti-proliferating cell nuclear antigen (PCNA; cat. no. sc-56), anti-Ki-67 (cat. no. sc-15402), anti-B-cell lymphoma 2 (Bcl-2; cat. no. sc-7382), anti-Bcl-2-associated X protein (Bax; cat. no. sc-6236) and anti-CD31 (cat. no. sc-71873) antibodies were all purchased from Santa Cruz Biotechnology, Inc. (Dallas, TX, USA). Anti-actin antibody, and goat anti-rabbit and goat anti-mouse immunoglobulin (Ig)G (cat. no. sc-66931) secondary antibodies were obtained from Santa Cruz Biotechnology, Inc.

### Irradiation

The exponentially growing cells were exposed at room temperature and irradiated with a Varian 600 CD X-ray linear accelerator (Varian Medical Systems, Inc., Palo Alto, CA, USA), at a dose rate of 2.5 Gy/min.

### Cell viability and colony formation assay

The exponentially growing cells were exposed at room temperature and a total dose of 6 Gy radiation was delivered in three fractions over three days. In the experimental study groups, 5 or 10 mM 3-MA was added to the cells for 2 h prior to irradiation. Six hours after the treatments, all of the cells were detached by trypsinization (Sigma-Aldrich) and the number of viable cells was counted. For the colony formation assay, the cells were seeded into six-well plates containing Dulbecco’s modified Eagle’s medium supplemented with 10% fetal bovine serum, and incubated for 14 days. The cells were fixed with ethanol (Cusabio Biotech Co., Ltd., Wuhan, China) and stained using 0.5% crystal violet (Sigma-Aldrich), while only colonies containing ≥50 cells were considered surviving colonies. The sensitizing enhancement ratio (SER) was calculated, according to the D_0_ values (dose of radiation producing a 37% survival rate), using the following formula: SER=D_0 untreated cells_/D_0 treated cells_.

### Cell cycle analysis

Flow cytometry was performed following DNA staining with propidium iodide (PI; Santa Cruz Biotechnology, Inc.), according to the manufacturer’s instructions. Briefly, the cells were harvested, washed with phosphate-buffered saline (PBS) and treated with 100 mg/ml RNase A (Santa Cruz Biotechnology, Inc.) for 30 min at room temperature. The cells were then stained with PI (1 mg/ml) solution at 4°C and incubated in the dark for 30 min. The cell cycle distribution was evaluated using a BD FACSArray™ Bioanalyzer system (BD Biosciences, San Jose, CA, USA).

### Apoptosis detection

The Annexin V-fluorescein isothiocyanate (FITC) Apoptosis Detection kit (Santa Cruz Biotechnology, Inc.) was used to assess the rate of cell apoptosis. Briefly, the untreated and treated cells were seeded in six-well plates and incubated for 24 h. The cells were then harvested, washed twice in PBS and stained with Annexin V-FITC and PI, according to the manufacturer’s instructions. Annexin V binds to apoptotic cells with exposed phosphatidylserine (early apoptosis), whereas PI labels cells with membrane damage (late apoptosis). The resulting fluorescence was detected by flow cytometry using CellQuest™ version 5.2.1 (BD Biosciences) analysis software.

### Western blot analysis

Protein extraction and tissue sample homogenization were performed as previously described (18,19). The cells were scraped into ice-cold PBS and centrifuged at 400 × g for 5 min at 4°C. The pelleted cells were then lysed in 50 ml boiling SDS solution (Sigma-Aldrich) and centrifuged at 4,350 × g for 5 min. Membrane protein was extracted from the resulting supernatant using Mem-PER Eukaryotic Membrane Protein Extraction kit (Pierce Biotechnology, Inc., Rockford, IL, USA). Protein concentrations were measured by NanoDrop 1000 (Thermo Fisher Scientific, Waltham, MA, USA) using bovine serum albumin (Santa Cruz Biotecnology, Inc.) as a standard. An equal amount of protein (8 *µ*g) was separated using 15% SDS-PAGE and transferred to polyvinylidene fluoride membranes. The membranes were then blocked with 5% skimmed milk (Cusabio Biotech Co., Ltd.) for 1 h and incubated overnight at 4°C with the following primary antibodies: anti-LC3-I/II (diluted 1:2,000), anti-beclin-1 (diluted 1:800), anti-VEGF (diluted 1:400), anti-caspase-3 (diluted 1:400), anti-caspase-9 (diluted 1:500), anti-PARP (diluted 1:400), anti-PCNA (diluted 1:1,000), anti-Ki-67 (diluted 1:800), anti-Bax (diluted 1:1,000) and anti-Bcl-2 (diluted 1:1,000). The membranes were then incubated with horseradish peroxidase-conjugated IgG secondary antibodies for 2 h at 37°C. The immunoreactive bands were visualized using an enhanced chemiluminescence system (Pierce Biotechnology, Inc.). To quantify equal loading, the membranes were re-probed with a primary antibody targeting β-actin.

### Xenograft experiment

The experiments of the present study followed the Declaration of Helsinki, and were approved by the institutional review board of Zhengzhou University Affiliation Cancer Hospital (Zhengzhou, China). The EC9706 cells (2.0×10^6^ cells) were injected subcutaneously into the lower right side flank of male athymic nude BALB/C-nu/nu mice (5–6 weeks-old; weighing 18–20 g), which were purchased from Keli China Experimental Animal Center (Beijing, China). The care and treatment of the mice were in accordance with institutional guidelines. When the tumor volume reached 100 mm^3^, six mice/group were treated with 3-MA (30 mg/kg intraperitoneally 1 h prior to radiation) and/or radiation (2 Gy/fraction, five fractions per week to equal a total dose of 20 Gy). The mice were divided into four treatment groups (n=12/group): Untreated, 3-MA alone, radiation alone and radiation combined with 3-MA. At the end of the experiments all of the mice were sacrificed by cervical dislocation. Tumor diameters were measured with calipers and tumor volume (V) was calculating using the following formula for a rotational ellipsoid: V=A×B^2^/2 (A, axial diameter; B, rotational diameter).

### Histological analysis of tumors

Tumor tissues derived from the four groups were fixed in 10% formalin (Cusabio Biotech Co., Ltd.), embedded in paraffin and cut into 5-*µ*m sections. Hematoxylin and eosin (H&E; Santa Cruz Biotechnology, Inc.) staining was performed on the tumor tissue for general morphological analysis. The samples were assayed for DNA fragmentation [terminal deoxynucleotidyl transferase dUTP nick end labeling (TUNEL) assay] using the *in situ* Cell Death Detection kit (Roche Molecular Biochemicals, Indianapolis, IN, USA). Briefly, following deparaffinization and dehydration, the tissue sections were incubated in proteinase K (DAKO North America, Inc., Carpinteria, CA, USA) for 15 min, washed with PBS, incubated in equilibration buffer and then in terminal deoxynucleotidyl transferase enzyme solution. The sections were subsequently rinsed in PBS, incubated with streptavidin-peroxidase conjugate (Sigma-Aldrich) and visualized using diaminobenzidine (Sigma-Aldrich), according to the manufacturer’s instructions.

### Measurement of tumor angiogenesis

Specific staining for endothelial cells was conducted using the neo-angiogenesis marker CD31. The slides were fixed using cold acetone (Cusabio Biotech Co., Ltd.) for 20 min. Following two washes with PBS, the tissue sections were incubated with 3% hydrogen peroxide (Santa Cruz Biotechnology, Inc.) in methanol for 30 min, in order to block endogeneous peroxidase activity. Primary antibody incubation was conducted at 37°C for 2 h, the slides were then incubated with goat anti-mouse IgG secondary antibody for 30 min, and with streptavidin biotin-peroxidase complex (Santa Cruz Biotechnology, Inc.) for 40 min. Following incubation with diaminobenzidine chromogen (Santa Cruz Biotechnology, Inc.), the tissue sections were re-stained with hematoxylin. Vessel density was determined by counting the number of microvessels per high-power field (Olympus IX 70; Olympus Corporation, Tokyo, Japan).

### Statistical analysis

All values are presented as the mean ± standard error of the mean. Statistical significance was analyzed by one-way analysis of variance with *post hoc* Dunnett’s test, using SPSS version 16.0 (SPSS, Inc., Chicago, IL, USA). P<0.05 was considered to indicate a statistically significant difference.

## Results

### Inhibition of autophagy increases radiosensitization of tumor cells in vitro

**Figure 1:**
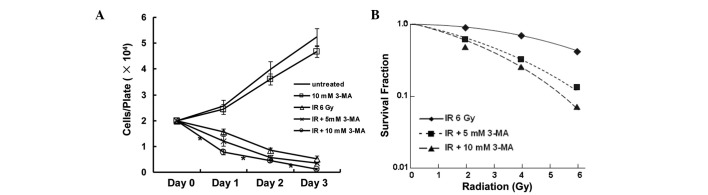
Inhibition of autophagy through 3-MA radiosensitizes tumor cells *in vitro*. (A) Cell proliferation was assessed by cell counting in the EC9706 human esophageal squamous carcinoma cell line. ^*^P<0.05 vs. IR alone. (B) Radiosensitivity was measured by colony formation assay. The results are the averages of triplicate samples. Error bars represent the standard error. 3-MA, 3-methyladenine; IR, ionizing radiation.

In order to demonstrate the enhancing effect of autophagy inhibition on radiosensitivity, cells were treated with 3-MA, which is a well-known inhibitor of autophagy in mammalian cells. Cell proliferation and colony formation were investigated in the EC9706 esophageal squamous carcinoma cell line. Treatment with 10 mM 3-MA alone led to a slight inhibition of cell growth. The viability of the cells was decreased in response to radiation, whereas a combination of radiation and 3-MA treatment markedly decreased the number of surviving cells (Fig. 1A). The radiosensitizing potential of 3-MA was ascertained by a clonogenic survival assay, and the SER was calculated based on the D_0_ values extrapolated from from the survival curves. The SER reached 1.76 when the cells were treated with a combination of 10 mM 3-MA and ionizing radiation (Fig. 1B). These results indicated that autophagy inhibition exhibits a radiosensitization potential *in vitro*.

### Irradiation induces autophagy in tumor cells

**Figure 2:**
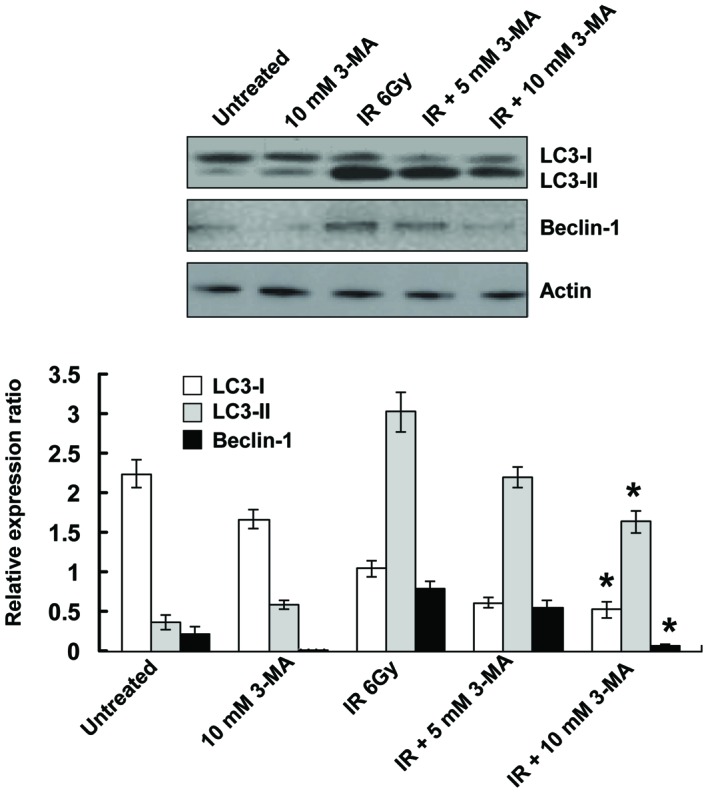
Induction of autophagy in EC9706 human esophageal squamous carcinoma cells by radiation. Cells were treated with irradiation and autophagic levels were detected. LC3 and beclin-1 protein expression levels were determined by western blot analysis. The ratios of LC3-I, LC3-II and beclin-1 to actin levels were determined densitometrically. The results are the averages of triplicate samples. Error bars represent the standard error. ^*^P<0.05 vs. IR alone. 3-MA, 3-methyladenine; IR, ionizing radiation; LC3, microtubule-associated protein light chain 3.

Autophagosome formation and expression of two essential autophagy-associated proteins, LC3 and beclin-1, were detected in the present study, in order to assess autophagy. During autophagy, phosphatidylethanolamine conjugates to the cytosolic form of LC3 (LC3-I), resulting in the formation of LC3-II. The amount of LC3-II is a commonly used indicator of autophagy (20). Beclin-1 participates in the early stages of autophagy, where it promotes the nucleation of the autophagic vesicle and recruits proteins from the cytosol (21). The present study detected an upregulation of beclin-1 protein expression levels and a downregulation of the conversion of LC3-I/II in EC9706 cells 24 h after co-treatment with radiation and 3-MA, thus indicating an increase in autophagic activity (Fig. 2). Following co-treatment of the cells with 3-MA, autophagic activity was downregulated; furthermore, decreased protein expression levels of beclin-1 and LC3-II were observed by western blotting. The expression levels of beclin-1 and LC3-II partly reverted to their original levels when the cells were treated with a combination of radiation and 10 mM 3-MA. These results provided evidence for the effective inhibitory effect of 3-MA on autophagy.

### Autophagy inhibition induces cell cycle arrest

**Figure 3:**
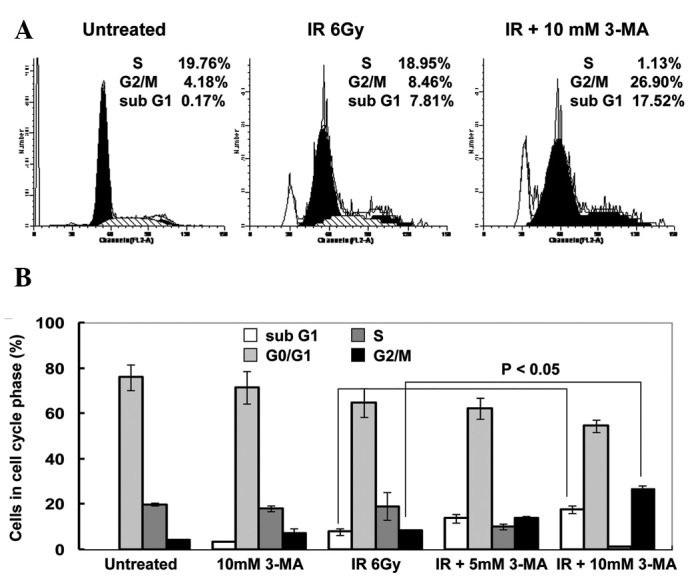
Inhibition of autophagy induces G_2_/M phase cell cycle arrest. (A) Effects of 3-MA, IR or their combination on cell cycle distribution of EC9706 human esophageal squamous carcinoma cells were assessed by flow cytometry. (B) Histograms of flow cytometric analysis. Cell populations with differential propidium iodide staining intensity were evaluated using CellQuest™ software. The results are the averages of triplicate samples. Error bars represent the standard error. 3-MA, 3-methyladenine; IR, ionizing radiation.

The possible effects of autophagy inhibition on the cell cycle distribution were investigated in the EC9706 cells treated with 3-MA, radiation, or their combination. Treatment of the cells with radiation (6 Gy) alone slightly affected the percentage of cells in each phase. In the presence of 3-MA, there was an increase in the number of EC9706 cells in G_2_/M phase by 65.7% and 218.0% following treatment with 5.0 and 10 mM of 3-MA, respectively. These results suggested that treatment with 3-MA resulted in a cell cycle arrest in G_2_/M phase, which is important for radiation sensitivity. Concomitant with the G_2_/M arrest was an elevation in the sub-G_1_ population, which is an indicator of apoptotic cell death (Fig. 3A and B). These results indicated that the effects of autophagy inhibition on radiation sensitization may be attributed to the induction of G_2_/M phase arrest and apoptosis.

### Autophagy inhibition increases cell apoptosis

**Figure 4:**
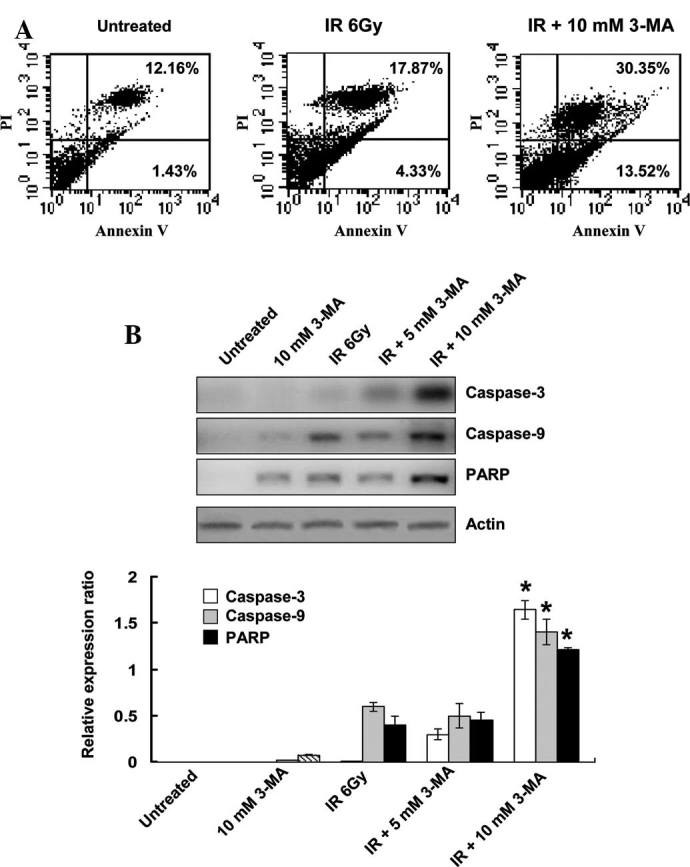
Autophagy inhibition increases the rate of apoptotic cell death induced by radiation. (A) Induction of apoptosis was analyzed using the Annexin-V assay and flow cytometry. In the dual parameter dot plots cells in early apoptosis are shown in the lower-right quadrant (Annexin V^+^/PI^−^), whereas cells in late apoptosis are shown in the upper right quadrant (Annexin V^+^/PI^+^). (B) Cell lysates were prepared and subjected to immunoblotting with antibodies against caspase-3, caspase-9 and PARP. The expression ratios in each experiment were determined densitometrically. The results are the averages of triplicate samples. Error bars represent the standard error.^*^P<0.05 vs. IR alone. 3-MA, 3-methyladenine; IR, ionizing radiation; PI, propidum iodide; PARP, poly(ADP ribose) polymerase.

To determine whether inhibition of autophagy was able to induce apoptosis, Annexin V-FITC and PI staining was conducted. Flow cytometry detected apoptotic cells 24 h after EC9706 cells were treated with radiation. When the irradiated cells were co-treated with 3-MA for 24 h, the number of Annexin V- and Annexin V/PI-positive cells significantly increased, as compared with that of cells treated with radiation alone (Fig. 4A). To further confirm the increase in apoptosis, the protein expression levels of the executioner caspases, caspase-3, caspase-9 and PARP, were determined. In the radiation-treated groups, caspase-3 and PARP were cleaved into their specific active forms, and their activity in the combined treatment group was significantly higher as compared with that in the cells treated with radiation alone (Fig. 4B). These results indicated that radiation-induced autophagy has a protective role in tumor cells against apoptosis, and inhibition of autophagy subsequently enhances the rate of apoptosis in the cells.

### Autophagy inhibition decreases VEGF protein expression levels

**Figure 5:**
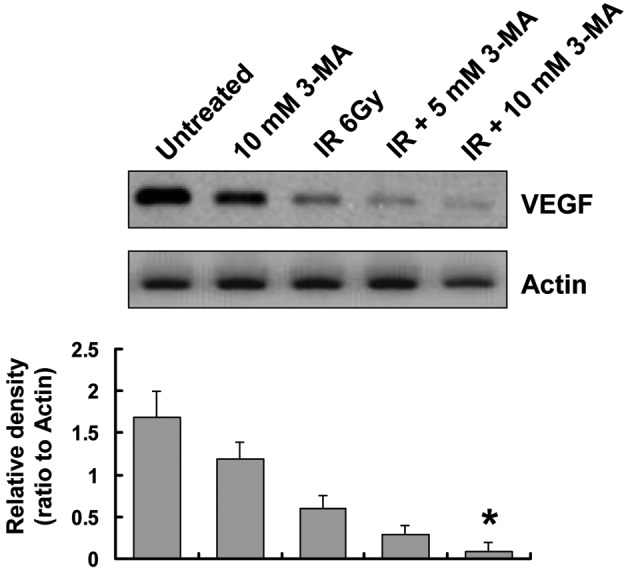
Effects of autophagy inhibition on the protein expression levels of VEGF. Western blot analysis demonstrated that VEGF protein expression levels were decreased when the EC9706 human esophageal squamous cell carcinoma cells were treated with 3-MA. The results are the averages of triplicate samples. Error bars represent the standard error.^*^P<0.05 vs. IR alone. 3-MA, 3-methyladenine; IR, ionizing radiation; VEGF, vascular endothelial growth factor.

To explore other potential mechanisms underlying the positive effects of autophagy inhibition on radiation in esophageal cancer, the role of autophagy in tumor angiogenesis was examined. VEGF is currently regarded as the most potent pro-angiogenic factor (22); therefore, the present study assessed VEGF protein expression levels in EC9706 cells. The protein expression levels of VEGF were reduced in EC9706 cells treated with 3-MA, as compared with those in the untreated cells. Of note, when irradiated cells were co-treated with 3-MA for 24 h, the protein expression levels of VEGF were significantly lower as compared with those in the radiation group (Fig. 5).

### Radiosensitizing effects of autophagy inhibition in vivo

**Figure 6:**
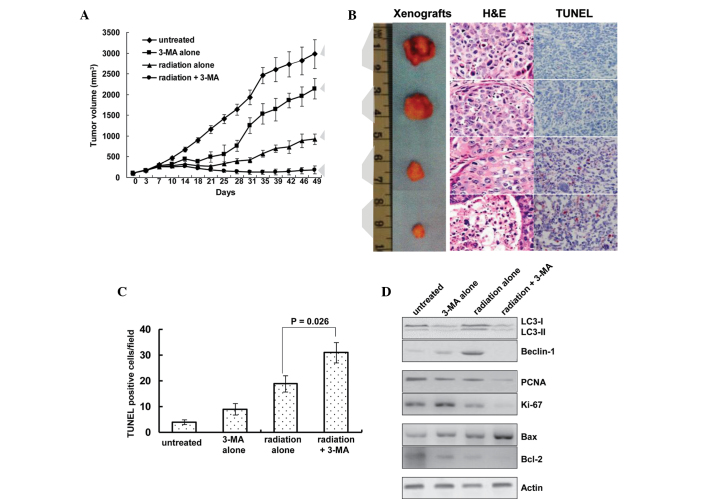
Autophagy inhibition sensitizes tumors to radiation *in vivo*. (A) Treatment with a combination of radiation and 3-MA reduced the growth of EC9706 human esophageal squamous cell carcinoma cell line xenografts. (B) Representative images of tumors and tumor slides subjected to TUNEL as well as H&E staining (magnification, ×400). Tumor size was decreased following treatment with 3-MA and radiation alone, and tumor size was smallest in the radiation + 3-MA group. (C) TUNEL-positive cells were quantified, showing increased numbers of apoptotic cells in single treated and significantly higher increases in the number of apoptotic cells in the combined treatment group. Results are expressed as the number of TUNEL-positive cells/field counted (five random fields per slide from a total of five slides per study group). (D) Western blot analysis of autophagic indicators LC-3 and beclin-1; proliferative indicators PCNA and Ki-67; and Bax and Bcl-2 apoptotic proteins in the xenografts. 3-MA, 3-methyladenine; IR, ionizing radiation; LC3, microtubule-associated protein light chain 3; PCNA, proliferating cell nuclear antigen; Bcl-2; B-cell lymphoma 2; Bax, Bcl-2-associated X protein; H&E, hematoxylin & eosin; TUNEL, terminal deoxynucleotidyl transferase dUTP nick end labeling.

The present study also investigated whether inhibition of autophagy was able to affect the tumor response to radiotherapy. The response of the EC9706 xenografts to radiation plus 3-MA was significantly enhanced, as compared with the that of the untreated, 3-MA alone and radiation alone groups (P<0.01, Fig. 6A). Treatment with 3-MA alone reduced the mean tumor volume by 28.2% (2,138.5±247.7 mm^3^, as compared with 2,977.3±352.2 mm^3^ in the untreated group), and treatment with radiation alone reduced the mean tumor volume by 68.29% (925.6±127.3 mm^3^, as compared with 2,977.3±352.2 mm^3^ in the untreated group). However, co-treatment of 3-MA with radiation resulted in significantly smaller tumors, with the tumor volume reduced by 93.9% (181.7±97.3 mm^3^, as compared with 2,977.3±352.2 mm^3^ in the untreated group; P<0.01).

Of note, the *in vitro* results were concordant with the findings of the *in vivo* experiments in the present study. A TUNEL assay was used to measure levels of apoptosis, and increased apoptosis was detected in the radiation plus 3-MA treatment group (Fig. 6B and C). The number of TUNEL-positive apoptotic cells per field was 4.1±0.9 in the untreated group, 9.3±2.2 in the 3-MA group, 18.9±3.1 in the radiation group and 31.2±3.9 in the co-treatment group (P<0.05).

Western blot analysis showed that radiation increased the protein expression levels of beclin-1 and LC3-II/LC3-I in the xenografts, whereas treatment with 3-MA inhibited the expression levels of LC3-II and beclin-1 (Fig. 6D). In addition, the protein expression levels of the proliferative markers PCNA and Ki-67 were assessed; in the tumors treated with radiation and 3-MA combined, the levels were decreased to a level that was barely detectable. Furthermore, the expression levels of the pro-apoptotic protein Bax and the anti-apoptotic protein Bcl-2 were detected. A significant upregulation in the protein expression levels of Bax and downregulation in the protein expression levels of Bcl-2 was detected in the tumors treated with radiation and 3-MA combined (Fig. 6C).

### Inhibition of tumor angiogenesis

**Figure 7:**
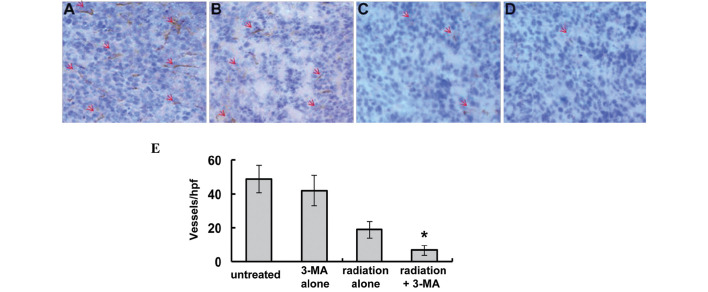
Inhibition of angiogenesis within xenograft tumors. Frozen tumor tissue sections from mice were stained with antibodies targeting CD31 (magnification, ×400). Sections from the (A) untreated; (B) 3-MA; (C) radiation; and (D) radiation + 3-MA groups are shown. Red arrows indicate microvessels. (E) Microvessel density in tumor tissue from the radiation plus 3-MA group was significantly decreased. ^*^P<0.05 vs. radiation alone. 3-MA, 3-methyladenine.

To investigate the effects of autophagy inhibition on tumor angiogenesis, immunohistochemical staining of frozen tumor tissue was performed using antibodies targeting CD31. Furthermore, angiogenesis within the tumor sections was evaluated by counting the number of microvessels in each section. Inhibition of autophagy by 3-MA suppressed angiogenesis, and mice in the radiation plus 3-MA treatment group exhibited a decreased microvessel density within the tumor, as compared with that in the radiation only group (Fig. 7; P<0.05).

## Discussion

Esophageal cancer is the eighth most common neoplastic malignancy worldwide, with 455,784 novel cases and 400,156 mortalities estimated in 2012, making it the sixth most common cause of cancer-associated mortality (23). Definitive radiotherapy and chemoradiation are the standard therapeutic approaches for the treatment of patients with esophageal cancer who are not suitable for surgery, due to the advanced stage of disease or significant co-morbidity (24). However, local treatment failure remains a major concern, with persistent or recurrent disease being reported in ~46–68% of patients, and most failures of local treatment occur when tumors are gross (25,26). Therefore, improvements in local control may translate into increased effectiveness in long-term cures. The clinical efficacy of radiotherapy is considered to be limited by normal tissue tolerance and inherent tumor radioresistance. Therefore, the development of novel radiosensitizing agents, which specifically sensitize tumor cells whilst protecting normal tissue function, is required.

Autophagy is an intracellular bulk degradation system, which is found ubiquitously in eukaryotes. Autophagy may lead to autophagic cell death through excessive self-digestion and degradation of essential cellular constituents under certain conditions (27). However, it has been suggested that the main role of autophagy is the assistance of cells in managing stressful metabolic environments, and thereby promoting cell survival (28). A family of autophagy-associated genes (ATG) is directly involved in the process of autophagy; LC3 is often used as a key molecule to monitor autophagosome formation in mammalian systems and beclin-1 is an essential modifier of the autophagic process (29). Furthermore, esophageal cancer cells may exploit autophagy to cope with the cytotoxicity of anti-cancer therapy. O’Donovan *et al* (18) investigated the cell-death mechanisms induced in esophageal cancer cells in response to the chemotherapeutic drugs 5-fuorouracil and cisplatin. In response to treatment, chemosensitive cell lines exhibited apoptosis, whereas chemoresistant cells exhibited autophagy. Inhibition of autophagy induction using small interfering (si)RNA targeted to beclin-1 and ATG7 significantly enhanced the effects of chemotherapeutic drugs, and reduced the recovery of drug-treated cells. Autophagy is frequently observed in cancer cells following exposure to ionizing radiation, and inhibition of autophagy has been shown to precipitate radiation-induced cell death (30). Lomonaco *et al* (31) previously demonstrated that γ-radiation activated autophagy and inhibition of autophagy significantly increased the radiosensitivity of glioma cells and glioma stem cells. Apel *et al* (32) investigated the effects of autophagy on the clonogenic survival of irradiated cancer cells, and showed that inhibition of autophagy-associated genes by specific target-siRNA oligonucleotides, led to enhanced cytotoxicity of radiotherapy in five types of human cancer cell lines. These results indicated that activation of autophagy under therapeutic stress contributes to the survival of cancer cells.

Whether autophagy contributes to tumor cell death or represents a radiation resistance mechanism in esophageal cancer has yet to be elucidated. The present study examined the contribution of radiation-induced autophagy using *in vitro* as well as *in vivo* models of esophageal cancer. Induction of autophagosome formation was confirmed by the protein expression of reliable markers of autophagy: LC3-II and beclin-1. Furthermore, treatment with the autophagy inhibitor 3-MA, which is a specific inhibitor of the early stage of the autophagic process, inhibited radiation-induced autophagy. Of note, treatment with a combination of radiation and 3-MA increased the therapeutic efficacy of radiation in human esophageal squamous cell carcinoma. Although the anti-cancer effects were limited in response to treatment with the various doses of radiation and 3-MA alone, cancer cell proliferation and tumor progression were markedly inhibited in the xenograft mouse model when the treatments were combined. These results suggested that autophagy represents a mechanism of resistance to radiation-mediated cell death.

The present study also aimed to determine the mechanisms underlying the effects of autophagy inhibition on radiosensitization in esophageal squamous cell carcinoma. The results of the present study demonstrated that treatment with 3-MA induced G_2_/M phase cell cycle arrest. It is well known that cancer cells are typically sensitive to radiation in G_2_/M phase. A key contributor to radiation resistance in autophagic cancer cells is their failure to engage in apoptosis (33). The flow cytometry results of the present study demonstrated that direct inhibition of autophagy by 3-MA significantly increased radiation-induced cell apoptosis, and this process was initiated through activation of caspases in esophageal squamous cell carcinoma cells. A TUNEL assay conducted on tumor tissue from xenografts showed that enhanced apoptosis was most pronounced in the radiation plus 3-MA treatment group. These findings were further confirmed by western blotting results, which demonstrated a significant upregulation in the protein expression levels of the pro-apoptotic protein Bax and downregulation in the protein expression levels of the anti-apoptotic protein Bcl-2. Furthermore, cellular proliferation was evaluated by measuring the expression levels of PCNA and Ki-67; decreased protein expression levels of PCNA and Ki-67 were most significant in the radiation plus 3-MA-treated tumor samples. These findings suggested that autophagy inhibition may enhance radiosensitization through increasing the rate of apoptosis and reducing tumor cell proliferation.

The complex association between autophagy and angio-genesis is currently poorly defined. Du *et al* (15) previously investigated the role of autophagy in angiogenesis. Treatment with 3-MA and siRNA targeting ATG5 were used to inhibit autophagy induced by nutrient deprivation of cultured bovine aortic endothelial cells. Inhibition of autophagy by 3-MA or siRNA targeting ATG5 suppressed angiogenesis, including VEGF-induced angiogenesis. Conversely, induction of autophagy by overexpression of ATG5 was able to promote angiogenesis in endothelial cells. It has previously been demonstrated that radiation-induced endothelial cell dysfunction may lead to impaired angiogenesis (34). The present study demonstrated that VEGF protein expression levels were decreased when autophagy was inhibited in esophageal squamous cell carcinoma cells following treatment with 3-MA, and analysis of tumor microvessels stained with rabbit anti-mouse CD31 antibody revealed that combining autophagy inhibition with radiation significantly reduced tumor microvessel density *in vivo*. These data indicated that autophagy inhibition synergistically enhances the anti-tumor activity of radiation through inhibition of tumor angiogenesis.

Autophagy inhibition has garnered attention as a novel anti-cancer therapeutic strategy, and inhibitors of autophagy have been reported to act as potent anti-cancer drugs and to sensitize cancer cells to anti-cancer therapy (35). The present study demonstrated that inhibition of autophagy was able to markedly enhance the anti-cancer effects of radiotherapy by promoting apoptotic cell death and downregulating angiogenesis. These results indicated that the use of anti-autophagy agents may improve the treatment outcomes of human esophageal squamous cell carcinoma.
